# UCSCXenaShiny: an R/CRAN package for interactive analysis of UCSC Xena data

**DOI:** 10.1093/bioinformatics/btab561

**Published:** 2021-07-29

**Authors:** Shixiang Wang, Yi Xiong, Longfei Zhao, Kai Gu, Yin Li, Fei Zhao, Jianfeng Li, Mingjie Wang, Haitao Wang, Ziyu Tao, Tao Wu, Yichao Zheng, Xuejun Li, Xue-Song Liu

**Affiliations:** School of Life Science and Technology, ShanghaiTech University, 201203 Shanghai, China; Shanghai Institute of Biochemistry and Cell Biology, Chinese Academy of Sciences, 200031 Shanghai, China; University of Chinese Academy of Sciences, 100049 Beijing, China; Department of Neurosurgery, Xiangya Hospital, Central South University, 410008 Changsha, China; Hunan International Scientific and Technological Cooperation Base of Brain Tumor Research, Xiangya Hospital, Central South University, 410008 Changsha, China; Xiangya School of Medicine, Central South University, 410013 Changsha, China; School of Pharmaceutical Sciences, Zhengzhou University, 450001 Zhengzhou, China; Roche Diagnostics (Shanghai) Limited, 201107 Shanghai, China; Department of Thoracic Surgery, Zhongshan Hospital, Fudan University, 200032 Shanghai, China; University of Chinese Academy of Sciences, 100049 Beijing, China; CAS Center for Excellence in Molecular Plant Sciences, 200032 Shanghai, China; State Key Laboratory of Medical Genomics, Shanghai Institute of Hematology, National Research Center for Translational Medicine, Rui-Jin Hospital, Shanghai Jiao Tong University, School of Medicine, 200025 Shanghai, China; School of Life Sciences and Biotechnology, Shanghai Jiao Tong University, 200240 Shanghai, China; State Key Laboratory of Medical Genomics, Shanghai Institute of Hematology, National Research Center for Translational Medicine, Rui-Jin Hospital, Shanghai Jiao Tong University, School of Medicine, 200025 Shanghai, China; Center for Precision Medicine Research and Training, Faculty of Health Sciences, University of Macau, 999087 Macau SAR, China; School of Life Science and Technology, ShanghaiTech University, 201203 Shanghai, China; School of Life Science and Technology, ShanghaiTech University, 201203 Shanghai, China; School of Pharmaceutical Sciences, Zhengzhou University, 450001 Zhengzhou, China; Department of Neurosurgery, Xiangya Hospital, Central South University, 410008 Changsha, China; Hunan International Scientific and Technological Cooperation Base of Brain Tumor Research, Xiangya Hospital, Central South University, 410008 Changsha, China; School of Life Science and Technology, ShanghaiTech University, 201203 Shanghai, China

## Abstract

**Summary:**

UCSC Xena platform provides huge amounts of processed cancer omics data from large cancer research projects (e.g. TCGA, CCLE and PCAWG) or individual research groups and enables unprecedented research opportunities. However, a graphical user interface-based tool for interactively analyzing UCSC Xena data and generating elegant plots is still lacking, especially for cancer researchers and clinicians with limited programming experience. Here, we present UCSCXenaShiny, an R Shiny package for quickly searching, downloading, exploring, analyzing and visualizing data from UCSC Xena data hubs. This tool could effectively promote the practical use of public data, and can serve as an important complement to the current Xena genomics explorer.

**Availability and implementation:**

UCSCXenaShiny is an open source R package under GPLv3 license and it is freely available at https://github.com/openbiox/UCSCXenaShiny or https://cran.r-project.org/package=UCSCXenaShiny. The docker image is available at https://hub.docker.com/r/shixiangwang/ucscxenashiny.

**Supplementary information:**

Supplementary data are available at *Bioinformatics* online.

## 1 Introduction

Over the past decade, large research programs including TCGA (The Cancer Genome Atlas) (Weinstein *et al.*, 2013), ICGC (International Cancer Genome Consortium) (Zhang *et al.*, 2011), PCAWG (Pan-cancer analysis of whole genomes) (Campbell *et al.*, 2020), GTEx (Genotype-Tissue Expression) (Ardlie *et al.*, 2015), CCLE (Cancer Cell Line Encyclopedia) (Barretina *et al.*, 2012) have generated large amounts of molecular data characterizing the different omics landscapes (including genomics, transcriptomics, proteomics and epigenomics) of thousands of tumors. The data have been uniformly preprocessed, curated and stored in data hubs of UCSC Xena (https://xenabrowser.net/datapages/) along with many public cancer datasets from individual research groups, providing unprecedented opportunities for either simple or systematic exploration of cancer behaviors and mechanisms at multiple molecular layers in individual cancer type or across cancer types (Goldman *et al.*, 2020).

Despite the fact that UCSC Xena provides a functional genomics explorer (https://xenabrowser.net/) to allow users to explore and analyze its multi-omics and clinical/phenotype data, it is still difficult for cancer researchers to rapidly explore all available UCSC Xena datasets, find what they need in their research, and download or analyze the corresponding data. Besides, the analysis features and visualization quality provided by UCSC Xena platform have room for improvement. Advanced functionalities for analyzing different molecular profiles from specified data hubs including TCGA, CCLE and PCAWG, and generating publication-ready result plots are still lacking.

In 2019, we developed UCSCXenaTools, an open-source R package for retrieving metadata and data from more than one thousand public UCSC Xena datasets (Wang and Liu, 2019). However, this package lacks analysis and visualization capabilities, and only provides a low-level application program interface (API) for accessing data. Thus, it is not suitable for cancer researchers with limited programming experience. Here, we are motivated to present UCSCXenaShiny, an R/CRAN package containing a web application based on the R Shiny framework (https://shiny.rstudio.com/) for quickly searching, retrieving, exploring, analyzing and visualizing data from UCSC Xena data hubs. This tool could effectively promote the practical use of UCSC Xena public data, and serve as an important complement to the functionality of current Xena functional genome explorer.

## 2 Tool description

**Fig. 1.**
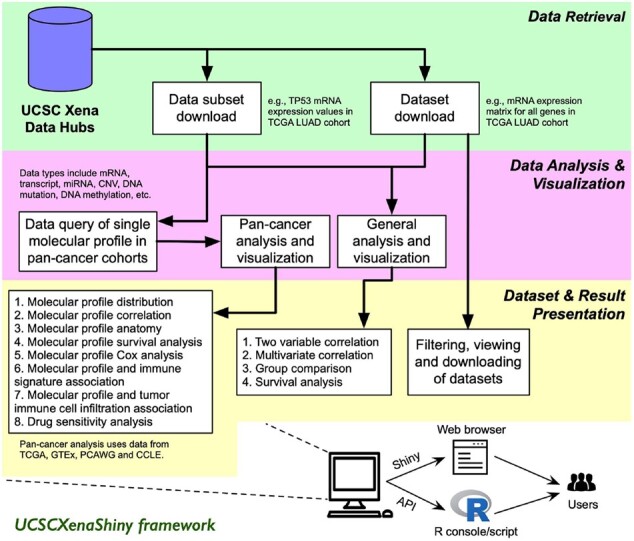
The architecture diagram of UCSCXenaShiny

UCSCXenaShiny uses both the R package interface (i.e. R functions) and the Shiny application interface to allow the user to efficiently retrieve and analyze data from UCSC Xena data hubs. The architecture of UCSCXenaShiny can be classified into three layers (Fig. 1). The first layer retrieves data from UCSC Xena data hubs and is built on the top of UCSCXenaTools (Wang and Liu, 2019). The second layer is implemented as an R package, it provides almost all core data and analysis features as built-in datasets and public functions (i.e. API) of the R package (Supplementary Table S1). The third layer is implemented as an R Shiny application and provides a graphical user interface for interactive exploration and analysis of UCSC Xena data. A demo of this Shiny is deployed at https://shiny.hiplot.com.cn/ucsc-xena-shiny/ for public use. UCSCXenaShiny has more functionalities compared with other UCSC Xena related tools (including UCSC Xena browser, UCSCXenaTools and xenaPython) (Supplementary Table S2).

### 2.1 R function interface

The public functions of UCSCXenaShiny can be divided into three categories based on their functionalities: (i) data retrieval; (ii) analysis and visualization of pan-cancer studies; (iii) advanced analysis and visualization (Supplementary Table S1).

### 2.2 Shiny interface

Its Shiny web application is the highlight of the UCSCXenaShiny software. It is a web-based software to provide interactive data retrieval, analysis and visualization for users. Similar to the R function interface, there are three core web pages: ‘Repository’, ‘General Analysis’ and ‘Quick PanCan Analysis’. The ‘Repository’ page contains a clickable table of UCSC Xena datasets, dataset filter widgets based on data hubs, cohorts, data types, keywords, etc., and corresponding action buttons (Supplementary Fig. S1). The current ‘General Analysis’ page contains four common analysis modules for exploring relationships between continuous variables, value difference between sample groups and survival curve difference between sample groups (Supplementary Fig. S2). The ‘Quick PanCan Analysis’ page contains several analysis modules for well-known pan-cancer data including TCGA, GTEx (Supplementary Fig. S3) and CCLE (Supplementary Fig. S4). For example: (i) comparisons of molecular profiles among samples, such as mRNA expression between TCGA tumor and normal control tissue (Supplementary Fig. S3A) or across different types of cancer (Maag, 2018) (Supplementary Fig. S3B) or CCLE cell lines (Supplementary Fig. S4A); (ii) association analysis between two molecular profiles with TCGA (Supplementary Fig. S3C) or CCLE (Supplementary Fig. S4B) data; (iii) association studies between a certain molecular profile and tumor/immune features, such as TMB (tumor mutational burden)/MSI (microsatellite instability)/stemness (Supplementary Fig. S3D) and immune gene signatures (Li *et al.*, 2020; Thorsson *et al.*, 2018) (Supplementary Fig. S3E); (iv) Kaplan–Meier survival analysis among samples with different levels of a molecular profile (Supplementary Fig. S3F); (v) association analysis between survival hazard ratio and a molecular profile with the Cox model across TCGA cancer types (Supplementary Fig. S3G); (vi) association analysis between mRNA expression of a gene (list) and cell line drug response (Supplementary Fig. S4C); (vii) exploring drug response differences between samples with different gene expression levels (Supplementary Fig. S4D).

## 3 Implementation

UCSCXenaShiny has been developed with R version ≥3.5 and Shiny following a modular and robust design of both R package and Shiny application. Continuous integration tests with CRAN R package is done automatically after each code commit to help test functionality and detect program bugs in a timely manner. Instructions on how to install, use UCSCXenaShiny and run the Shiny application are presented in the public GitHub repository (https://github.com/openbiox/UCSCXenaShiny). A detailed manual of built-in data and public R functions are organized and described in the package reference page (https://openbiox.github.io/UCSCXenaShiny/reference/index.html). Instructions including texts and videos on how to use functionalities of the Shiny application are documented in the help page of the Shiny application. Tooltips are adopted to help users to understand and customize the parameter setting. The Shiny application also shows data table behind each result plot and provides data download buttons to facilitate the archiving of data and result reproducibility.

## 4 Conclusion

In recent years, several bioinformatics platforms or tools, such as cBioPortal (Cerami *et al.*, 2012), Genomic Data Commons (GDC) data portal (Grossman *et al.*, 2016), ICGC Data Portal (Zhang *et al.*, 2019), CVCDAP (Guan *et al.*, 2020) and UCSC Xena (Goldman *et al.*, 2020) have been constructed for the analysis and visualization of cancer genomics data (Supplementary Table S3). UCSCXenaShiny works as a UCSC Xena client, cBioportal, ICGC data portal, GDC data portal are independent data portals. Compared with these other data portals, UCSC Xena platform is featured with a comprehensive collection of public cancer genome datasets, and combined analysis between public and researchers’ own data (Goldman *et al.*, 2020). However, UCSC Xena only provided limited number of analysis tools. For efficient cancer genome data download, integration, exploration and visualization, we built UCSCXenaShiny to allow a wide range of users to perform interactive analysis of UCSC Xena data by either programming or graphical interface operation. Since its release, UCSCXenaShiny has been downloaded for more than 10 000 times around the world (according to the API for CRAN package download counts, from the RStudio CRAN mirror, https://cranlogs.r-pkg.org/). We believe that UCSCXenaShiny could effectively promote the practical use of public cancer data and serve as an important complement to the functionality of current Xena functional genome explorer.

## Supplementary Material

btab561_Supplementary_Data
