# Supplementary material for: UCSCXenaShiny: an R/CRAN package for interactive analysis of UCSC Xena data
Source: Bioinformatics. 2021 Jul 29;38(2):527–9. doi: 10.1093/bioinformatics/btab561 (PMC8723150; doi:10.1093/bioinformatics/btab561)
Supplement: btab561_Supplementary_Data [file btab561_supplementary_data.pdf]

Table S1

Data Query

Functions to query data from UCSC Xena data hubs in single molecular level. The functionality of functions below may overlap, we rank them by importance.

`query_molecule_value()`	Get Molecule or Signature Data Values from Dense (Genomic) Matrix Dataset of UCSC Xena Data Hubs
`query_pancan_value()`	Query Single Identifier or Signature Value from Pan-cancer Database
`get_ccle_cn_value()` `get_ccle_gene_value()` `get_ccle_protein_value()` `get_ccle_mutation_status()` `get_pancan_value()` `get_pancan_gene_value()` `get_pancan_transcript_value()` `get_pancan_protein_value()` `get_pancan_mutation_status()` `get_pancan_cn_value()` `get_pancan_methylation_value()` `get_pancan_miRNA_value()`	Fetch Identifier Value from Pan-cancer Dataset
`query_toil_value_df()` `query_toil_value_df()`	Obtain ToilHub Info for Single Molecule
`available_hosts()`	Show Available Hosts

Analysis and Visualiation for Pan-Cancer Study

Functions to analyze and visualize data mainly from TCGA and CCLE

`tcga_surv_get()` `tcga_surv_plot()`	TCGA Survival Analysis
`analyze_gene_drug_response_asso()`	Analyze Association between Gene (Signature) and Drug Response with CCLE Data
`vis_gene_drug_response_asso()`	Visualize Gene and Drug-Target Association with CCLE Data
`analyze_gene_drug_response_diff()`	Analyze Difference of Drug Response (IC50 Value (uM)) between Gene (Signature) High and Low Expression with CCLE Data
`vis_gene_drug_response_diff()`	Visualize Gene and Drug Response Difference with CCLE Data
`vis_ccle_tpm()`	Visualize CCLE Gene Expression
`vis_ccle_gene_cor()`	Visualize CCLE Gene Expression Correlation
`vis_gene_TIL_cor()`	Heatmap for Correlation between Gene and Tumor Immune Infiltration (TIL)
`vis_gene_immune_cor()`	Heatmap for Correlation between Gene and Immune Signatures
`vis_gene_cor()`	Visualize Gene-Gene Correlation in TCGA
`vis_gene_cor_cancer()`	Visualize Gene-Gene Correlation in a TCGA Cancer Type
`vis_gene_stemness_cor()`	Visualize Correlation between Gene and Tumor Stemness
`vis_gene_tmb_cor()`	Visualize Correlation between Gene and TMB (Tumor Mutation Burden)
`vis_gene_msi_cor()`	Visualize Correlation between Gene and MSI (Microsatellite instability)
`vis_pancan_anatomy()`	Visualize Single Gene Expression in Anatomy Location
`vis_toil_TvsN()`	Visualize Pan-cancer TPM (tumor (TCGA) vs Normal (TCGA & GTEx))
`vis_toil_TvsN_cancer()`	Visualize Gene TPM in Single Cancer Type (Tumor (TCGA) vs Normal (TCGA & GTEx))
`vis_unicox_tree()`	Visualize Single Gene Univariable Cox Result from Toil Data Hub

General Analysis and Visualiation

Functions to analyze and visualize general data, i.e., any valid data from UCSC Xena or users

`vis_identifier_cor()`	Visualize Identifier-Identifier Correlation
`vis_identifier_multi_cor()`	Visualize Correlation for Multiple Identifiers
`vis_identifier_grp_comparison()`	Visualize Comparison of an Molecule Identifier between Groups
`vis_identifier_grp_surv()`	Visualize Identifier Group Survival Difference
`ezcor()`	Run Correlation between Two Variables and Support Group by a Variable
`ezcor_batch()`	Run correlation between two variables in a batch mode and support group by a variable
`ezcor_partial_cor()`	Run partial correlation

Table S1. Major functions provided by *UCSCXenaShiny*. All public data and functions provided by the tool is available at https://openbiox.github.io/UCSCXenaShiny/reference/index.html.

Table S2

Tool		UCSCXenaShiny	UCSC Xena Browser	UCSCXenaTools	xenaPython
	Visual download	Yes	Yes (limited)	No	No
Data download features	Any dataset download	Yes	Yes	Yes	No
	Any data subset download	Yes	Yes	Yes	Yes
	TCGA/CCLE data quick query	Yes	No	No	No
	Plot data download	Yes	Yes	No	No
General analysis features	Scatter correlation (for two variables)	Yes	Yes	No	No
	Correlation heatmap (for multiple variables)	Yes	No	No	No
	Group comparison	Yes	Yes	No	No
	Survival analysis	Yes	Yes	No	No
	Genomic signatures	Yes	Yes	No	No
	Sample filtering	Yes	Yes	No	No
	Custom data support	Yes	Yes	No	No
	Visual spreadsheet	No	Yes	No	No
Specified pan-cancer analysis features	TCGA/GTEx, PCAWG, CCLE molecular profile value distribution	Yes	No	No	No
	TCGA, PCAWG, CCLE molecular profile correlation	Yes	No	No	No
	CCLE drug sensitivity analysis	Yes	No	No	No
	TCGA/GTEx molecular profile anatomy	Yes	No	No	No
	TCGA, PCAWG molecular profile survival analysis	Yes	Yes (limited)	No	No
	TCGA molecular profile and immune signature/infiltration association	Yes	No	No	No
	TCGA, PCAWG molecular profile and tumor features (stemness/MSI/TMB) association	Yes	No	No	No
Generate publication ready plots		Yes	No	No	No
Has API		Yes (available in self-constructed R package)	Yes (available as UCSCXenaTools and xenaPython)	It's itself an API	It's itself an API
Target user type		User with and without programming background	User without programming background	User with programming background	User with programming background
URL		https://github.com/openbiox/UCSCXenaShiny	https://xenabrowser.net/	https://github.com/ropensci/UCSCXenaTools	https://github.com/ucscXena/xenaPython

Table S2. A comparison of different tools for retrieving and analysis of UCSC Xena's data. Note: "API" means application programming interface. "URL" means uniform resource locator.

Figure S1

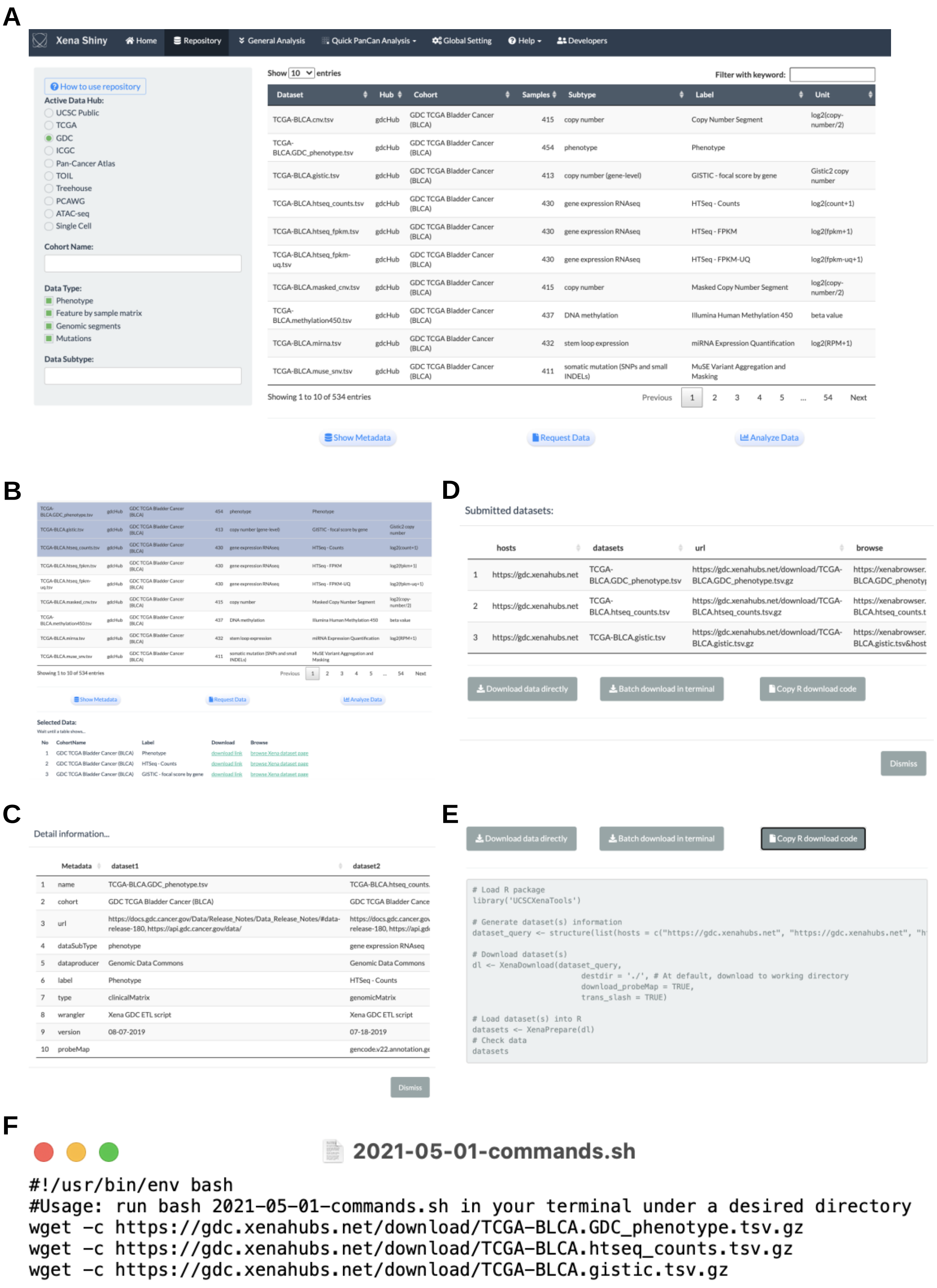
A
Xena Shiny
Home
Repository
General Analysis
Quick PanCan Analysis
Global Setting
Help
Developers
How to use repository
Active Data Hub:
UCSC Public
TCGA
GDC
ICGC
Pan-Cancer Atlas
TOIL
Treehouse
PCAWG
ATAC-seq
Single Cell
Cohort Name:
Data Type:
Phenotype
Feature by sample matrix
Genomic segments
Mutations
Data Subtype:
Show 10 entries
Filter with keyword:
Dataset | Hub | Cohort | Samples | Subtype | Label | Unit
TCGA-BLCA.cnv.tsv | gdcHub | GDC TCGA Bladder Cancer (BLCA) | 415 | copy number | Copy Number Segment | log2(copy-number/2)
TCGA-BLCA.GDC_phenotype.tsv | gdcHub | GDC TCGA Bladder Cancer (BLCA) | 454 | phenotype | Phenotype
TCGA-BLCA.gistic.tsv | gdcHub | GDC TCGA Bladder Cancer (BLCA) | 413 | copy number (gene-level) | GISTIC - focal score by gene | Gistic2 copy number
TCGA-BLCA.htseq_counts.tsv | gdcHub | GDC TCGA Bladder Cancer (BLCA) | 430 | gene expression RNAseq | HTSeq - Counts | log2(count+1)
TCGA-BLCA.htseq_fpkm.tsv | gdcHub | GDC TCGA Bladder Cancer (BLCA) | 430 | gene expression RNAseq | HTSeq - FPKM | log2(fpkm+1)
TCGA-BLCA.htseq_fpkm-uq.tsv | gdcHub | GDC TCGA Bladder Cancer (BLCA) | 430 | gene expression RNAseq | HTSeq - FPKM-UQ | log2(fpkm-uq+1)
TCGA-BLCA.masked_cnv.tsv | gdcHub | GDC TCGA Bladder Cancer (BLCA) | 415 | copy number | Masked Copy Number Segment | log2(copy-number/2)
TCGA-BLCA.methylation450.tsv | gdcHub | GDC TCGA Bladder Cancer (BLCA) | 437 | DNA methylation | Illumina Human Methylation 450 | beta value
TCGA-BLCA.mirna.tsv | gdcHub | GDC TCGA Bladder Cancer (BLCA) | 432 | stem loop expression | miRNA Expression Quantification | log2(RPM+1)
TCGA-BLCA.muse_snv.tsv | gdcHub | GDC TCGA Bladder Cancer (BLCA) | 411 | somatic mutation (SNPs and small INDELs) | MuSE Variant Aggregation and Masking
Showing 1 to 10 of 534 entries
Previous 1 2 3 4 5 ... 54 Next
Show Metadata
Request Data
Analyze Data
B
Selected Data:
C
Detail information...
Metadata | dataset1 | dataset2
1 name | TCGA-BLCA.GDC_phenotype.tsv | TCGA-BLCA.htseq_counts.
2 cohort | GDC TCGA Bladder Cancer (BLCA) | GDC TCGA Bladder Cance
3 url | https://docs.gdc.cancer.gov/Data/Release_Notes/Data_Release_Notes/#data-release-180, https://api.gdc.cancer.gov/data/ | https://docs.gdc.cancer.gov release-180, https://api.gd
4 dataSubType | phenotype | gene expression RNAseq
5 dataproducer | Genomic Data Commons | Genomic Data Commons
6 label | Phenotype | HTSeq - Counts
7 type | clinicalMatrix | genomicMatrix
8 wrangler | Xena GDC ETL script | Xena GDC ETL script
9 version | 08-07-2019 | 07-18-2019
10 probeMap | | gencode.v22.annotation.ge
Dismiss
D
Submitted datasets:
hosts | datasets | url | browse
1 https://gdc.xenahubs.net | TCGA-BLCA.GDC_phenotype.tsv | https://gdc.xenahubs.net/download/TCGA-BLCA.GDC_phenotype.tsv.gz | https://xenabrowser. BLCA.GDC_phenoty
2 https://gdc.xenahubs.net | TCGA-BLCA.htseq_counts.tsv | https://gdc.xenahubs.net/download/TCGA-BLCA.htseq_counts.tsv.gz | https://xenabrowser. BLCA.htseq_counts.t
3 https://gdc.xenahubs.net | TCGA-BLCA.gistic.tsv | https://gdc.xenahubs.net/download/TCGA-BLCA.gistic.tsv.gz | https://xenabrowser. BLCA.gistic.tsv&host
Download data directly
Batch download in terminal
Copy R download code
Dismiss
E
Download data directly
Batch download in terminal
Copy R download code
# Load R package
library('UCSCXenaTools')
# Generate dataset(s) information
dataset_query <- structure(list(hosts = c("https://gdc.xenahubs.net", "https://gdc.xenahubs.net", "h
# Download dataset(s)
dl <- XenaDownload(dataset_query,
destdir = './', # At default, download to working directory
download_probeMap = TRUE,
trans_slash = TRUE)
# Load dataset(s) into R
datasets <- XenaPrepare(dl)
# Check data
datasets
F
2021-05-01-commands.sh
#!/usr/bin/env bash
#Usage: run bash 2021-05-01-commands.sh in your terminal under a desired directory
wget -c https://gdc.xenahubs.net/download/TCGA-BLCA.GDC_phenotype.tsv.gz
wget -c https://gdc.xenahubs.net/download/TCGA-BLCA.htseq_counts.tsv.gz
wget -c https://gdc.xenahubs.net/download/TCGA-BLCA.gistic.tsv.gz

Figure S1. Major features provided by “Repository” page of Shiny application of *UCSCXenaShiny*. (A) Interface of “Repository” page. The table on the main panel shows current available datasets. The filters on the left panel provide functionality to filter datasets. The three buttons shown on the bottom of the main panel are used to operate datasets selected by users. (B) How to select specified datasets. The rows in dataset table are clickable. When a row is selected, metadata of the corresponding dataset will be queried from UCSC Xena data hubs, and the download link and the link to observe the dataset in UCSC Xena browser are provided. (C) When the button “Show Metadata” is clicked, all important metadata of datasets are shown. (D) When the button “Request Data” is clicked, links of datasets are shown and three buttons are provided to download datasets. (E) When the button “Copy R download code” is clicked, the code will be shown to tell users how to download the selected datasets by R. (F) When the button “Batch download in terminal” is clicked, a Shell script containing download code with wget command is automatically downloaded by the user browser so that the user can run this script in terminal. See **Figure S4** for analyzing data after clicking the button “Analyze Data”.

Figure S2

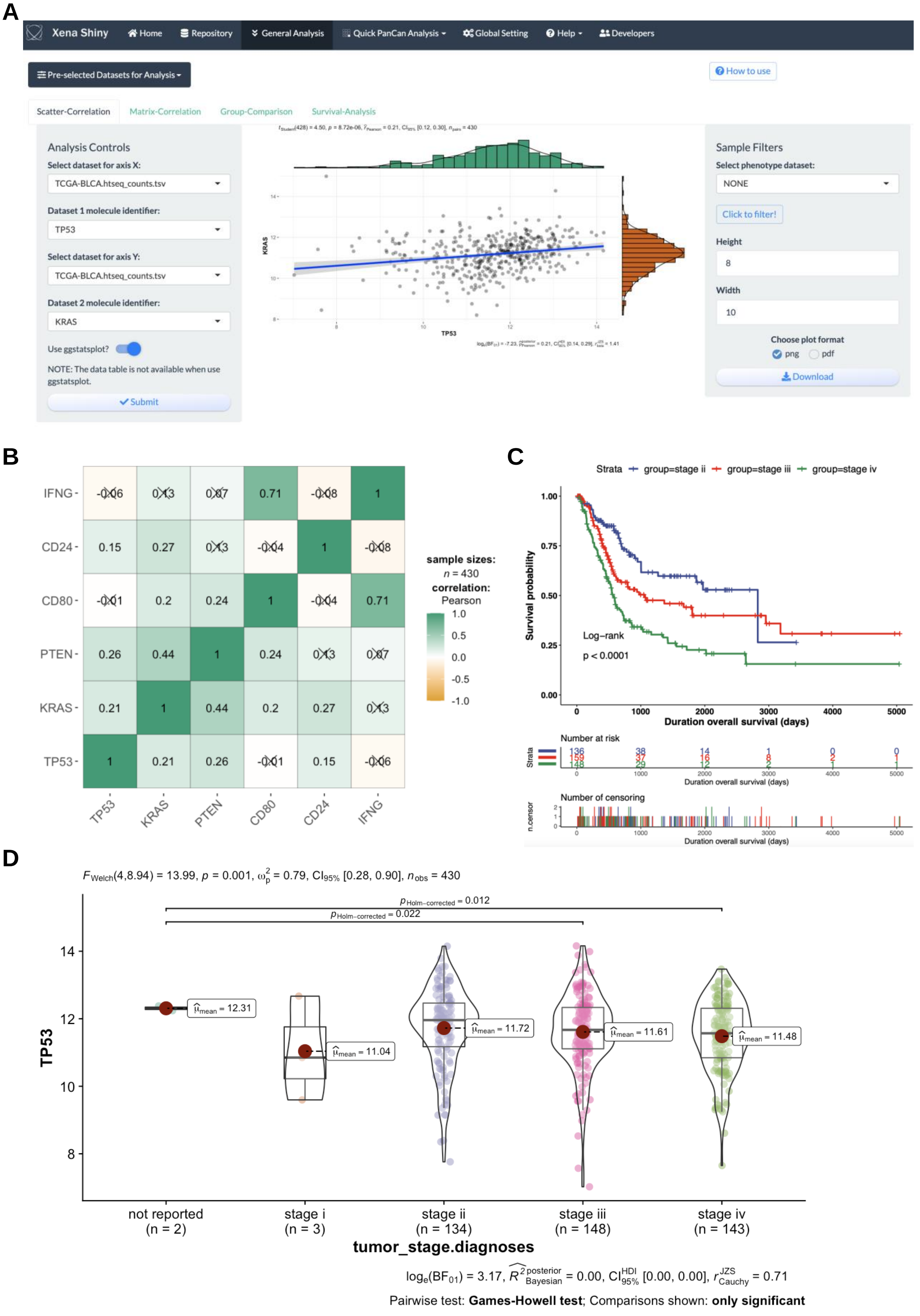
A
Xena Shiny
Home
Repository
General Analysis
Quick PanCan Analysis
Global Setting
Help
Developers
Pre-selected Datasets for Analysis
How to use
Scatter-Correlation
Matrix-Correlation
Group-Comparison
Survival-Analysis
Analysis Controls
Select dataset for axis X:
TCGA-BLCA.htseq_counts.tsv
Dataset 1 molecule identifier:
TP53
Select dataset for axis Y:
TCGA-BLCA.htseq_counts.tsv
Dataset 2 molecule identifier:
KRAS
Use ggstatsplot?
NOTE: The data table is not available when use ggstatsplot.
Submit
KRAS
TP53
Sample Filters
Select phenotype dataset:
NONE
Click to filter!
Height
8
Width
10
Choose plot format
png
pdf
Download
B
IFNG
CD24
CD80
PTEN
KRAS
TP53
sample sizes:
n = 430
correlation:
Pearson
1.0
0.5
0.0
-0.5
-1.0
C
Strata
group=stage ii
group=stage iii
group=stage iv
Survival probability
Log-rank
p < 0.0001
Duration overall survival (days)
Number at risk
Number of censoring
D
TP53
not reported (n = 2)
stage i (n = 3)
stage ii (n = 134)
stage iii (n = 148)
stage iv (n = 143)
tumor_stage.diagnoses
Pairwise test: Games-Howell test; Comparisons shown: only significant

Figure S2. Features provided by "General Analysis" page of Shiny application of *UCSCXenaShiny*. Any dataset with proper format in UCSC Xena data hubs can be used for the current 4 supported analysis modules. (A) "Scatter-Correlation" module is used for exploring correlation between two continuous variables. The UI (user interface) widgets on the left and right sides of plot region are used for controlling analysis, visualization, sample filtering and result outputting. (B) "Matrix-Correlation" module is used for exploring correlation along multiple continuous variables. (C) "Survival-Analysis" module is used for exploring survival difference between sample groups. Here a continuous variable (e.g., mRNA expression of a gene) is supported to set sample groups by a threshold. (D) "Group-Comparison" module is used for exploring and visualizing value difference of a continuous variable between sample groups. The datasets used for demo analyses in this figure are selected shown as **Figure S3**.

Figure S3

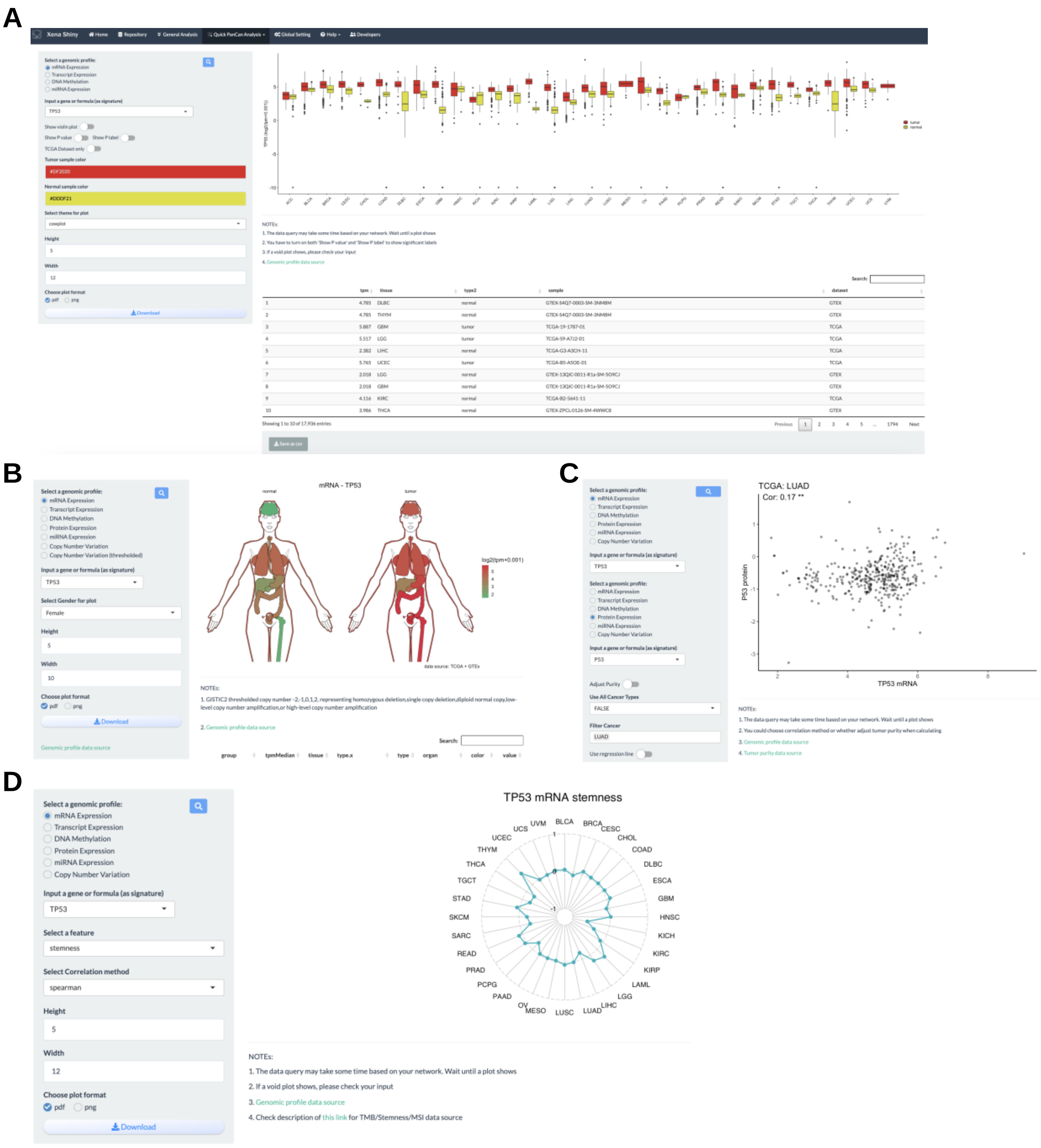
A
B
mRNA - TP53
normal
tumor
log2(tpm+0.001)
data source: TCGA + GTEx
C
TCGA: LUAD
Cor: 0.17 **
P53 protein
TP53 mRNA
D
Select a genomic profile:
mRNA Expression
Transcript Expression
DNA Methylation
Protein Expression
miRNA Expression
Copy Number Variation
Input a gene or formula (as signature)
TP53
Select a feature
stemness
Select Correlation method
spearman
Height
5
Width
12
Choose plot format
pdf
png
Download
TP53 mRNA stemness
UVM
BLCA
BRCA
CESC
CHOL
COAD
DLBC
ESCA
GBM
HNSC
KICH
KIRC
KIRP
LAML
LGG
LIHC
LUAD
LUSC
MESO
OV
PAAD
PCPG
PRAD
READ
SARC
SKCM
STAD
TGCT
THCA
THYM
UCEC
UCS
NOTEs:
1. The data query may take some time based on your network. Wait until a plot shows
2. If a void plot shows, please check your input
3. Genomic profile data source
4. Check description of this link for TMB/Stemness/MSI data source

Figure S3 *continued*

E

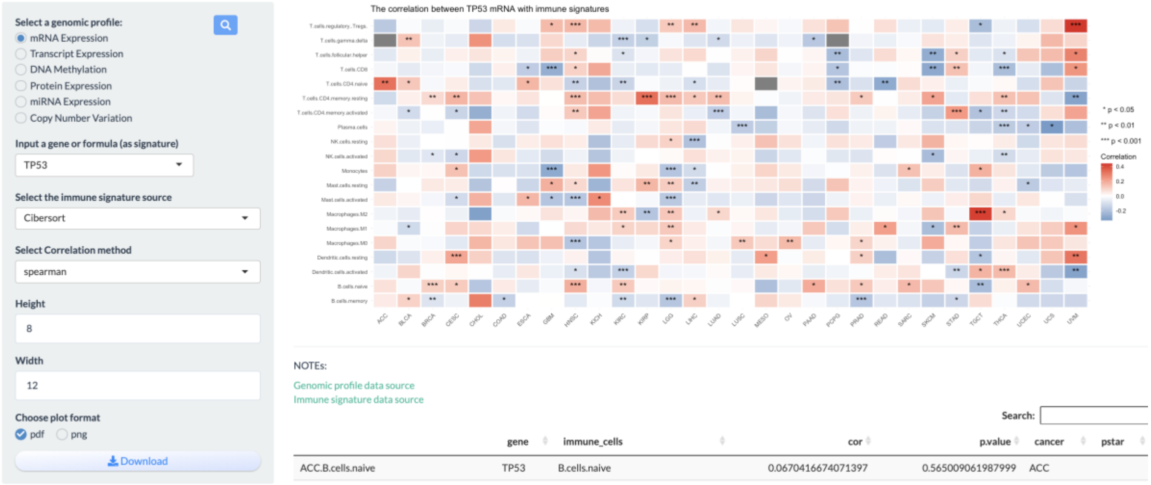

F

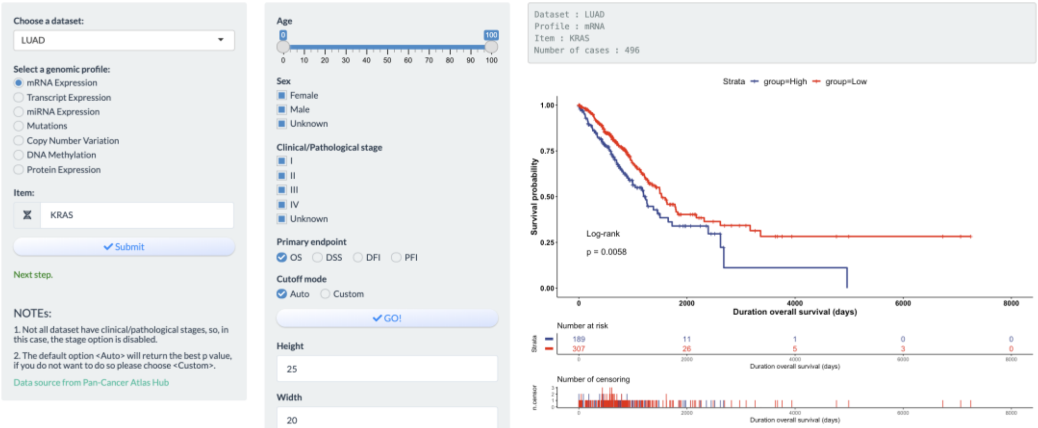

G

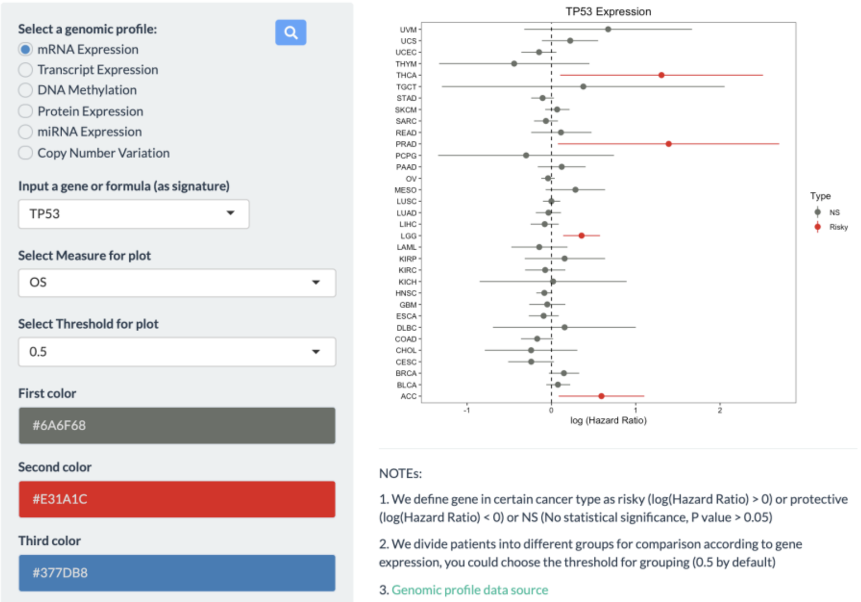

Figure S3. Analysis features with TCGA/GTEx data provided by “Quick PanCan Analysis” page of Shiny application of *UCSCXenaShiny*. The analysis modules contains panels for parameters and outputting setting, visualization and data presentation. (A) The module showing molecular profile distribution, value difference between tumor and normal tissue across TCGA cancer types. (B) The module showing molecular profile distribution, value difference (between tumor and normal tissue) across tumor tissue origins. (C) The module showing correlation between two molecular profiles. (D) The module showing association between molecular profile and tumor features including stemness, TMB (tumor mutational burden) and MSI (microsatellite instability). (E) The module showing association between molecular profile and tumor immune signatures or immune cell subsets. (F) The module showing survival difference between sample groups corresponding different molecular profile levels in TCGA pan-cancer or specified tumor type. In this sub-plot, TCGA LUAD cohort is selected. (G) The module showing association between survival risk and molecular profile level across TCGA cancer types with univariable Cox models. NOTE: Some analysis pages are too large to show all the details.

Figure S4

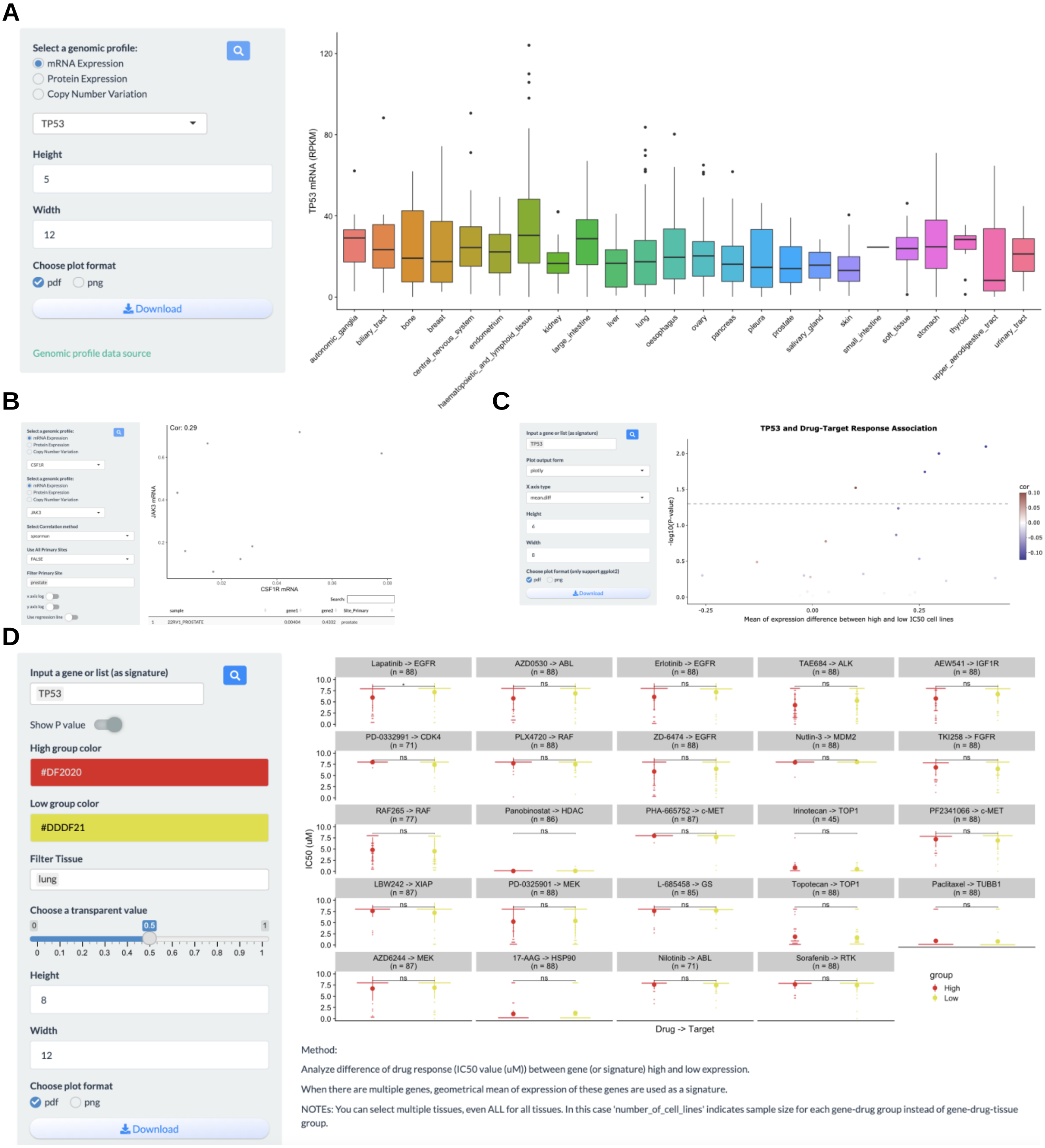

A
Select a genomic profile:
mRNA Expression
Protein Expression
Copy Number Variation
TP53
Height
5
Width
12
Choose plot format
pdf
png
Download
Genomic profile data source
TP53 mRNA (RPKM)
autonomic_ganglia
biliary_tract
bone
breast
central_nervous_system
endometrium
haematopoietic_and_lymphoid_tissue
kidney
large_intestine
liver
lung
oesophagus
ovary
pancreas
pleura
prostate
salivary_gland
skin
small_intestine
soft_tissue
stomach
thyroid
upper_aerodigestive_tract
urinary_tract
B
Cor: 0.29
CSF1R mRNA
JAK3 mRNA
C
TP53 and Drug-Target Response Association
-log10(P-value)
Mean of expression difference between high and low IC50 cell lines
cor
D
Input a gene or list (as signature)
TP53
Show P value
High group color
#DF2020
Low group color
#DDDF21
Filter Tissue
lung
Choose a transparent value
Height
8
Width
12
Choose plot format
pdf
png
Download
Lapatinib -> EGFR (n = 88)
AZD0530 -> ABL (n = 88)
Erlotinib -> EGFR (n = 88)
TAE684 -> ALK (n = 88)
AEW541 -> IGF1R (n = 88)
PD-0332991 -> CDK4 (n = 71)
PLX4720 -> RAF (n = 88)
ZD-6474 -> EGFR (n = 88)
Nutlin-3 -> MDM2 (n = 88)
TKI258 -> FGFR (n = 88)
RAF265 -> RAF (n = 77)
Panobinostat -> HDAC (n = 86)
PHA-665752 -> c-MET (n = 87)
Irinotecan -> TOP1 (n = 45)
PF2341066 -> c-MET (n = 88)
LBW242 -> XIAP (n = 87)
PD-0325901 -> MEK (n = 88)
L-685458 -> GS (n = 85)
Topotecan -> TOP1 (n = 88)
Paclitaxel -> TUBB1 (n = 88)
AZD6244 -> MEK (n = 87)
17-AAG -> HSP90 (n = 88)
Nilotinib -> ABL (n = 71)
Sorafenib -> RTK (n = 88)
IC50 (uM)
Drug -> Target
group
High
Low
Method:
Analyze difference of drug response (IC50 value (uM)) between gene (or signature) high and low expression.
When there are multiple genes, geometrical mean of expression of these genes are used as a signature.
NOTEs: You can select multiple tissues, even ALL for all tissues. In this case 'number_of_cell_lines' indicates sample size for each gene-drug group instead of gene-drug-tissue group.

Figure S4. Analysis features with CCLE cancer cell line data provided by “Quick PanCan Analysis” page of Shiny application of *UCSCXenaShiny*. The analysis modules contains panels for parameters and outputting setting, visualization and data presentation. (A) The module showing molecular profile value distribution across CCLE cancer cell line tissue origins. (B) The module showing correlation between two molecular profiles. (C) The module showing association between gene mRNA expression and tumor drug response. (D) The module showing drug response IC50 value difference between low and high expression of a gene (use expression median as cutoff). NOTE: Some analysis pages are too large to show all the details.

Table S3

	Purpose/key feature	Downside
**UCSCXenaShiny**	An R package with both command caller and web GUI for interactively exploring UCSC Xena data.	It depends on R environment and has many dependent packages to support its analysis and visualization features.
**UCSC Xena Browser**	An online tool to explore functional genomic data sets for correlations between genomic and/or phenotypic variables.	1) It can not be used to download datasets in batch or download a dataset subset. 2) It has no specific analysis and visualization modules for the most commonly used pan-cancer (TCGA, PCAWG, etc.) cohorts.
**UCSCXenaTools**	An R API to access both a dataset and a dataset subset from UCSC Xena data hubs.	It has no analysis and visualization features for UCSC Xena datasets.
**xenaPython**	A Python API to access a dataset subset from UCSC Xena data hubs.	1) It can not be used to download a whole UCSC Xena dataset. 2) It has no analysis and visualization features for UCSC Xena datasets.
**cBioPortal/GDC portal/ICGC portal**	Web resources for exploring multidimensional cancer genomics data.	They mainly act as cancer genomics data providers. Their analysis and visualization features are limited.
**CVCDAP**	Seamless molecular and clinical analysis of a single TCGA cohort or comparison of two TCGA cohorts with relevance.	1) It cannot be used to download UCSC data. 2) Its analysis features are limited to TCGA datasets. 3) For user custom data, it only supports mutation analysis.

Table S3. Summary and comparison of the purpose/key feature and downside of UCSC Xena related tools (UCSCXenaShiny, UCSC Xena Browser, UCSCXenaTools, xenaPython) and other similar tools (cBioPortal, GDC portal, ICGC portal, CVCDAP).
